# Relationship of Body Mass Index and Footprint Morphology to the Actual Height of the Medial Longitudinal Arch of the Foot

**DOI:** 10.3390/ijerph18189815

**Published:** 2021-09-17

**Authors:** Carolina Rosende-Bautista, Pedro V. Munuera-Martínez, Teresa Seoane-Pillado, María Reina-Bueno, Francisco Alonso-Tajes, Sergio Pérez-García, Gabriel Domínguez-Maldonado

**Affiliations:** 1 Department of Health Sciences, University of A Coruña, 15403 Ferrol, Spain; carolina.rosende@udc.es (C.R.-B.); francisco.alonso.tajes@udc.es (F.A.-T.); sergio.perez.garcia@udc.es (S.P.-G.); 2 Department of Podiatry, University of Seville, 41009 Seville, Spain; pmunuera@us.es (P.V.M.-M.); gdominguez@us.es (G.D.-M.); 3 Preventive Medicine and Public Health Unit, Department of Health Sciences, University of A Coruña-INIBIC, 15006 A Coruña, Spain; maria.teresa.seoane.pillado@udc.es

**Keywords:** foot morphology, medial longitudinal arch, foot posture index, footprint, body mass index

## Abstract

The medial longitudinal arch height of the foot is linked to individual characteristics such as sex and body mass index, and these characteristics have been shown to be associated with conditions such as flat feet. In this cross-sectional descriptive study, we examined the medial longitudinal arch morphology in an adult population to determine if there are differences related to sex and body mass index, and values were obtained for the foot posture index. Normalized anthropometric measurements and arch indices were calculated from footprints. Groups, defined by sex and body mass index, were compared, and the correlations between body mass index and the variables were determined. In the population studied (266 women and 177 men), significant differences between men and women for the foot posture index and normalized arch measurements were found. Analysis of the variables related to body mass index indicated there were significant differences in arch indices. Significant differences and positive correlations were also found between the arch index and body mass index for the left and right feet among the men and women studied. The results obtained allow us to reflect on and analyze whether the medial longitudinal arch morphology classification methods used in the clinical and research setting are adequate or whether the influence of factors such as body mass index can generate confusion.

## 1. Introduction

Studies of flat feet [1,2,3] have linked medial longitudinal arch (MLA) morphology to individual characteristics such as sex, race, age, foot dimensions, and body mass index (BMI). Recent systematic revisions and meta-analyses [4,5], however, have revealed there is no internationally agreed-upon clinical method for classifying MLA height (not involving the use of ionizing radiation), making it difficult to draw conclusions from evidence linking different MLA morphologies with individual characteristics, and with foot and lower limb conditions.

Physiological characteristics, such as sexual dimorphism of the pelvis and lower limbs and sexually determined differences in ligament laxity and joint mobility [6], can affect MLA morphology. Differences in foot morphology between the sexes have been corroborated by studies aimed at improving the design of footwear [7,8] and anthropometric measurements using radiographic imaging [9]. Nevertheless, other studies involving clinical measurements of MLA morphology [10,11,12,13] have not found these differences between the sexes.

Flat foot studies point to BMI as an associated factor [3,14,15], but the alteration of footprint morphology caused by increases in BMI [16] can cause confusion if these morphology alterations are used as the sole diagnostic method for assessing MLA height. Song et al. [17] concluded that the reduction in height that is indicated by the footprint does not reflect the same reductions in the bone structure.

One of the most common methods used in several studies [18,19] to classify MLA height is to observe and measure footprint morphology. This method has been used in several studies [20,21] that report a correlation between measurements of MLA bone structure and footprint morphology.

In another study, anthropometric measurements of MLA bone structure are described, and their validity was analyzed to determine MLA height in contrast to radiographic measurements [22]. Evans et al. [23] and McPoil et al. [24] concluded that anthropometric measurements are a valid and reliable method for clinical practice and research. In these applications, anthropometric measurements have been limited by the lack of reference ranges, as well as the complexity of the devices used in the studies.

The objectives of the present study were to describe MLA morphology in an adult population using three measurement techniques and to establish whether differences in the values that determine MLA are connected to sex and BMI.

To determine the relevance of MLA morphology in the diagnosis and treatment of biomechanical pathologies of the foot, the influence of factors such as sex and BMI need to be accounted for, as well as whether the methods used to classify MLA height may be influenced by these factors. This would allow us to determine if an analyzed factor, such as excess weight, is really a causal factor for MLA alterations, or if the method used to perform the classification is influenced by that factor.

## 2. Materials and Methods

### 2.1. Research Study Design and Sample Selection

A cross-sectional descriptive study using non-probabilistic convenience sampling was carried out between October 2017 and June 2018. Participants included patients (61.5% *n* = 260), their companions (22.9% *n* = 97), and students (15.6% *n* = 66) at the University Clinic of Podiatry in Ferrol (University of A Coruña) and the Clinical Area for Podiatry of the Nursing, Physiotherapy and Podiatry Department at the University of Seville. Informed consent was given by all participants in this study, which was reported on favorably by the Research Ethics Committee of Galicia (registry code 2015/516).

For inclusion in the study, participants were required to be over 18 years of age, regardless of foot type. Criteria for exclusion included serious foot or leg injuries (fractures, pathological processes in the acute phase, etc.), serious neurological or joint conditions or diseases (paralysis, ankylosis, etc.), congenital lower limb malformation or deformity (club foot, etc.), or previous foot surgery; the initial interviews allowed us to not recruit people affected by these pathologies.

Of the 423 individuals that took part in the study, 50% were estimated to have morphological alterations to their MLAs, with a confidence level of 95% and ±5% accuracy, and assuming a 10% loss.

### 2.2. Data Collect

At both locations (Ferrol and Sevilla), data were collected by a single observer with over 15 years of experience in foot evaluation.

The socio-demographic variables of sex and age were obtained, along with anthropometric variables for weight (kg) and height (cm).

All foot measurements and assessments were performed on subjects in a bipedal posture at their own angle and base while walking, which allowed for the most natural distribution of load for the individual. The values used were an average of three measurements made in the same exploratory act by a single observer.

The fact that anthropometric measurement techniques are, clinically, the least used in our profession led us to conduct a reliability test of the anthropometric measurement method for 122 feet, and the intraclass correlation coefficient (ICC) was calculated for the intraobserver and interobserver, as well as the average differences between measurements, using 95% estimated confidence intervals (CIs).

Two observers took anthropometric measurements from 61 people using the methodology described above. Observer 1 took each measurement twice, with 7–10 days of separation between measurements. Observer 2 took the measurements once, coinciding with the first measurements taken by Observer 1, in a different examination room.

### 2.3. Foot Posture

To **determine foot posture** while standing, the validated scale for clinical use, the Foot Posture Index 6 (FPI-6), was used, which was defined by Redmond et al. [25]. Patient assessments and classifications of foot posture were performed following the FPI-6 user guide. The FPI-6 values obtained for each foot (−12 to +12) were treated as continuous variables and as categorical variables, according to the guide’s instructions.

### 2.4. Anthropometric Measurement of MLA

The normalized anthropometric measurements of MLA height were taken according to a specific methodology that was identical for all participants, similar to that previously used by Williams et al. [26], McPoil et al. [24]., and Evans et al. [23].

Following the methodology described by Mall et al. [27], the navicular tubercle and the distal medial end of the first metatarsal were marked. Once the anatomical points of reference were located, measurements were taken (with the patient standing) using a millimeter-marked foot sizer and two millimeter-marked rulers (set square and triangle), whose shapes allowed them to be placed stably on the transverse plane for accurate measurements on the sagittal plane. The following variables were measured: total foot length (from the back of the heel to the most distal point on the longest toe), truncated length (length from the back of the heel to the most distal point on the head of the first metatarsal), navicular tubercle height (height from the ground to the lowest point of the navicular tubercle), and height of the bridge to 50% of the total length (height of the bridge of the foot at the point where it coincides longitudinally with 50% of the total length).

Once the arch height measurements were obtained, the values were normalized to the different lengths, and the following variables were defined:

***Bony arch index (BAI):*** navicular tubercle height/total length;
***Bony arch index truncated (BAIT):*** navicular tubercle height/truncated length;
***Arch height index (AHI):*** bridge height 50% length/length.

### 2.5. Footprint Measurement

The Arch Index (AI) was the method chosen to measure and classify footprint morphology. Footprints were obtained using a manual ink pedigraph, digitalized, and measured using AutoCAD software (Autodesk Inc., San Rafael, CA, USA), following the methodology defined by Cavanagh and Rogers [28]. They were then classified, according to the values obtained, as cavus footprints when the AI was <0.21, as normal when the AI was 0.21–0.26, and as flat when the AI was >0.26.

### 2.6. Statistical Analysis

A univariate analysis was performed describing the qualitative variables as absolute values and percentages. The quantitative variables are reported as average values ± standard deviation, median, and range.

The possible associations between the qualitative variables were checked using the chi-squared test, or Fisher’s exact test. The average values were compared using the unpaired Student’s *t*-test when two groups were considered, and the ANOVA test for more than two groups. Non-parametric, Mann–Whitney *U*, and Kolmogorov–Smirnov contrasts were used for numerical variables not conforming to normal distributions. Correlations between BMI and the quantitative variables of MLA morphology were analyzed, and the Spearman’s ρ was calculated using IBM SPSS Statistics 22 software (IMB, Armonk, NY, USA) and Epidat version 3.1 (Dirección Xeral de Innovación e Xestión da Saúde Pública, Xunta de Galicia and Pan American Health Organization—PAHO-WHO).

## 3. Results

Of the 423 individuals included in the study, 63.03% were women. The average age was 44.4 ± 18.9, and 252 of the individuals (59.6%) were obese or overweight.

A significant link between sex and categorized BMI was identified, with 53% of the women being overweight or obese vs. 70.7% of the men (Appendix A, Table A1).

Appendix A, Table A2 presents the values for the variables related to foot posture, MLA morphology, and footprint measurements.

The average MLA variable measures were found to be similar for both left and right feet. According to the FPI-6 classification, for the left limb, 41.5% of the feet were normally positioned, followed by 38.2% of the feet that were found to be slightly or markedly overpronated, and 20.4% that were slightly or markedly oversupinated. Similar percentages were obtained for the right limb (44.4% normal; 36.6% slightly or markedly overpronated; 18.9% slightly or markedly oversupinated).

AI categorization revealed that the most common footprints fell within the values of a normal arch for both feet (left foot = 38.6%; right foot = 43.6%).

In the reliability test of the normalized anthropometric measurements, the values obtained were found to have good or very good concordance in the intraobserver tests—ICC (BAI) = 0.92; ICC (AHI) = 0.87; ICC (BAIT) = 0.94—and good concordance in the interobserver tests—ICC (BAI) = 0.78; ICC (AHI) = 0.79; ICC (BAIT) = 0.73. See Appendix A, Table A3.

### 3.1. Analysis of Sex-Related Differences

Significant differences were found in the average FPI-6 values between men and women, with the men having markedly lower values than the women for both feet (left foot: 2.5 ± 4.9 vs. 3.9 ± 4,5; right foot: 2.6 ± 5.0 vs. 3.7 ± 4.2). Significant differences were also identified in the total and normalized anthropometric measurements (Appendix A, Table A4). It should be noted, however, that no significant differences were found in the average AI values, and that categorization of the variable did show a significant link with the sex of the patient in both feet. For the left side, 46.5% of the men had normal feet and 20.4% had a high arch, while 34.0% of the women had normal feet and 30.9% had a high arch. For the right side, among the men, 42.0% had normal feet and 41.4% had a high arch. The percentages for the women were, respectively, 44.5% and 30.9% (Appendix A, Table A4).

The lengths and heights showed significantly higher average values for men compared to women. Normalizing height did not eliminate these differences between the sexes, which were significant for the BAI, AHI, and BAIT (Appendix A, Table A4).

### 3.2. Analysis of BMI-Related Differences

Analysis of the variables registered for BMI revealed no significant differences between the groups in either the FPI-6 or normalized MLA measurements. The average AI values were significantly higher (*p* < 0.001, Appendix A, Table A5) in the group of participants who were obese (0.27 ± 0.048) and overweight (0.25 ± 0.057) compared to the group with normal weight (0.22 ± 0.056).

Analysis by sex revealed statistically significant differences in the group of men for AI values and no significant differences for the categorized AI. Among the women, the differences were significant in both feet for the BAI, BAIT, and AI values, and the categorized AI. No significant differences in the FPI-6 values were found, nor were they found in the categorization of FPI-6 for either of the sexes (Appendix A, Table A6).

A positive correlation was found between the BMI and AI values among men (r = 0.201, *p* = 0.011 for the left foot and r = 0.187, *p* = 0.019 for the right foot). This correlation was also found in women (r = 0.463, *p* < 0.001 for the left foot and r = 0.441, *p* < 0.001 for the right foot). A linear correlation was found for men between the BAI for both feet (*r* = 0.003, *p* = 0.973 for the left foot and r = −0.0017, *p* = 0.834 for the right foot), while the BAIT variables (r =0.019, *p* = 0.816 for the left foot and r = −0.0017, *p* = 0.834 for the right foot) were negative and significant only in the women: BAI (r = −0.160, *p* = 0.009 left foot and r = −0.162, *p* = 0.008 right foot) and BAIT (r = −0.161, *p* = 0.009 left foot and r = −0.168, *p* = 0.006 right foot (Appendix A, Table A6).

## 4. Discussion

This study analyzed MLA characteristics in a population that was diverse in age and podiatric health. The average values obtained were close to the average values that have been reported in previous studies [24,29] carried out on similarly diverse populations, even though, in this study, non-probability sampling was carried out. The fact that our sampling included subjects with and without foot disorders and of different ages likely allows the results in some parameters to be consistent with other studies.

The results indicate sex-related differences in MLA height. The women studied were found to have a greater tendency for overpronation and lower normalized MLA height measurements than the men studied. Footprint morphology did not reflect these differences, which may be due to the influence of other factors that affect footprint morphology, such as BMI.

The significant differences in average FPI-6 values between the sexes, with a higher average value found among the women than the men (Appendix A, Table A4), which was undetected in other studies [12,25], could be explained by the physiological characteristics of women (greater ligament laxity, greater range of articular movement, and lesser muscular strength) and sexual dimorphism (wider pelvis, altered Q angle, and tendency towards genu valgum among women), which affect MLA morphology.

As in previous studies [12,30,31], no statistical significance or correlation was found between the FPI-6 values and the different BMI groups among the men or the women.

The methods used for MLA measurements were found to have good interobserver and intraobserver reliability (Appendix A, Table A2), which were lower than those used in previous studies by Butler et al. [10] and Mc Poil et al. [24].

The average values obtained from the population were close to those referenced by McPoil et al. [24]. Significant differences between the sexes were found for both feet in both absolute and normalized values (Table A4), with longer feet and higher MLA identified in men. These results accord with those reported by Hashimoto et al. [9], who used radiographic measurements, and Zaho et al. [32], who used 3D scanner measurements, and results from studies on footwear design [7,8], which have all pointed to existing differences between the sexes in MLA contour. Additionally, these results accord with those presented by Mc Poil et al. [24] regarding the AHI. These differences between the sexes were not identified in the studies of physically active populations with asymptomatic feet by Butler et al. [10], Zifchock et al. [11], Wunderlich and Cavanagh [7], or Xiong et al. [13]. The heterogeneous nature of the population for this study may explain the novel results not reported in previous studies whose populations were selected for certain characteristics.

Regarding the relation between MLA height and BMI, the results obtained report important discrepancies when comparing different methods of measurement. The FPI-6 and AHI values were not significantly different between either different BMI groups or across the sexes. The BAI and BAIT values were found to have differences only in the group of women, and the AI appeared to be a parameter positively correlated with BMI in women and men. Consequently, we believe caution should be applied when interpreting results relating to MLA morphology and obesity when the parameter for categorizing MLA height was obtained using the AI or footprints. This is because, as observed in this study, other measurements do not indicate alterations of MLA height in groups categorized by BMI. Based on these results, we consider that in the obese and overweight populations, the MLA measurements of the footprint may not be valid to classify morphology. We should consider in future research if other measurements to classify the morphology of the ALI through the footprint are also influenced by BMI.

In the study, the analysis of BAI, AHI, and BAIT values in groups categorized by BMI showed different results according to sex. Among men, BMI had no influence on these values and no significant correlations were found. Among women, normalized navicular tubercle height values (BAI and BAIT) were found to have differences according to BMI, and a significant non-linear negative correlation between BAI and BAIT variables with BMI was identified (R-values ranged between −0.160 and −0.168, Appendix A, Table A6).

The average AI values for men and women were not significantly different between the sexes, which is in agreement with the results of previous footprint analysis studies [3,13,18]. In AI categorization, differences between the sexes appeared, which included discrepancies according to laterality (Appendix A, Table A4).

The AI values, when grouped by BMI, had (for both feet and both sexes) significant differences between groups, with a clear increase in AIs occurring in individuals with higher BMIs. Correlation analysis confirmed a significant positive correlation between AIs and BMIs in both sexes, with R-values found to be higher among women (Appendix A, Table A6). The AI categorization of BMI groups (with significant differences found only among women) suggests that low arch morphology is more common among obese individuals.

One limitation of this study was that while collecting data, measurements taken from individuals in a natural posture who were chosen for their being accustomed to clinical exploration could introduce attention bias (if feeling watched, subjects may change their postures). This may negatively affect the results obtained in the reliability test for the normalized anthropometric measurements. Furthermore, differences in MLA morphology between the sexes could have been shown more clearly if data had been obtained regarding physiological factors, morphological characteristics of the lower limbs, and the degree of physical activity, to limit possible confounding bias.

## 5. Conclusions

In the population studied, significant differences were found between men and women for FPI-6 values and normalized arch measurements. Analysis of the variables related to BMI indicated that there were significant differences between the sexes for the arch index. Significant differences and positive correlations were also found between the arch index and body mass index for the left and right foot among men and women. The results obtained allow us to reflect on whether the MLA morphological classification methods commonly used individually in clinical and research settings are adequate for establishing the categorization of MLA morphology or whether the existence of influences of factors intrinsic to the individual, such as sex or BMI, can lead us to establish the need to always perform the classification of the morphology of the MLA according to the results obtained with two or more methods.

Future studies should determine the most reliable and valid method for the measurement of MLA height during clinical exploration. Given the results obtained, we propose that normalized anthropometric measurements might be the clinical technique that allows us to classify the morphology of the ALI with greater rigor.

## Appendix A

**Table A1:** Sociodemographic and anthropometric characteristics of the participants.

Characteristics	All Subjects (*n* = 423)		Men 157 (36.96%)	Women 266 (63.03%)	*p*
	*n* (%)	Mean ± SD	Mean ± SD	Mean ± SD	
**Age (years)**		44.43 ± 18.87	45.20 ± 18.17	43.98 ± 19.29	0.357
**Weight (kg)**		73.94 ± 15.12	82.83 ± 12.86	68.71 ± 13.87	<0.001
**Height (m)**		1.65 ± 0.09	1.73 ± 0.07	1.60 ± 0.07	<0.001
**BMI (kg/m^2^)**		27.10 ± 5.21	27.66 ± 4.34	26.77 ± 5.66	0.073
**BMI SCORE**			***n* (%)**	***n* (%)**	
**Underweight**	2 (0.5)		0	2 (0.8)	0.002
**Normal Weight**	169 (40.0)		46 (29.3)	123 (46.2)	
**Overweight**	147 (34.8)		70 (44.6)	77 (28.9)	
**Obesity**	105 (24.8)		41 (26.1)	64 (24.1)	

Underweight: (body mass index) BMI ≤ 18.5 kg/m^2^; normal weight: 18.5 kg/m^2^ ≤ BMI < 25 kg/m^2^; overweight: 25 kg/m^2^ ≤ BMI < 30 kg/m^2^; obesity ≥ 30 kg/m^2^.

**Table A2:** Descriptive values of Foot Posture Index 6, anthropometric measurements of MLA and arch index on both feet.

Measurements		Left Foot	Right Foot		
		Mean ± SD	Median (Rank)	Mean ± SD	Median (Rank)
**FPI-6**		3.35 ± 4.70	4 (−9–12)	3.31 ± 4.56	4 (−12 + 12)
**Navicular tubercle height (cm)**		3.96 ± 0.78	4.00 (1.50–6.50)	4.12 ± 0.76	4.1 (1.50–6.80)
**Total foot length (cm)**		24.92 ± 1.61	24.80 (21.40–29.30)	24.91 ± 1.61	24.8 (21.4–29.30)
**Truncated foot length (cm)**		18.08 ± 1.24	18 (15.00–21.00)	18.19 ± 1.23	18 (15–22)
**Dorsal height 50% length (cm)**		5.77 ± 0.62	5.8 (4.00–8.10)	5.80 ± 0.58	5.8 (4.20–7.80)
**BAI**		0.15 ± 0.02	0.15 (0.06–0.25)	0.16 ± 0.02	0.16 (0.06–0.27)
**AHI**		0.23 ± 0.02	0.23 (0.16–0.32)	0.23 ± 0.01	0.23 (0.17–0.31)
**BAIT**		0.39 ± 0.03	0.40 (0.27–0.52)	0.39 ± 0.03	0.39 (0.30–0.52)
**AI**		0.24 ± 0.06	0.25 (0.00–0.38)	0.24 ± 0.06	0.25 (0.03–0.39)
**SCORE**		***n* (%)**	***n* (%)**		
**FPI-6**	**Neutral (0–+5)**	175 (41.5%)	188 (44.4%)		
	**Pronated (+6–+9)**	118 (28%)	122 (28.8%)		
	**Highly pronated (+10–+12)**	43 (10.2%)	33 (7.8%)		
	**Supinated (−1–−4)**	61 (14.5%)	57 (13.5%)		
	**Highly supinated (−5–−12)**	25 (5.9%)	23 (5.4%)		
**AI**	**Normal arch (0.21–0.26)**	163 (38.6%)	184 (43.6%)		
	**Flat arch (>0.26)**	145 (34.4%)	147 (34.8%)		
	**Cavus arch (<0.21)**	114 (27%)	91 (21.6%)		

**FPI-6**: Foot Posture Index 6; **BAI**: Bony arch index; **AHI**: Arch height index; **BAI**: Bony arch index truncated; **AI**: Arch Index.

**Table A3:** Intraobserver and interobserver reliability test of the anthropometric measurement.

	Intraobserver		Interobserver	
	SEM (95% CI)	ICC (95%)	SEM (95% CI)	ICC (95%CI)
**BAI**	0.0058 (0.0050; 0.0065)	0.9217 (0.8899–0.9446)	0.0122 (0.0107–0.0137)	0.7822 (0.7026–0.8425)
**AHI**	0.0049 (0.004; 0.0057)	0.8687 (0.8175–0.9063)	0.0076 (0.0065–0.0086)	0.7932 (0.7170–0.8507)
**BAIT**	0.0079 (0.0069; 0.0089)	0.9409 (0.9167–0.9583)	0.0186 (0.0162–0.0209)	0.7326 (0.6385–0.8051)

**SME**: Standard error of measurements. **BAI**: bony arch index = navicular tubercle height/total foot length; **AHI**: arch height index = dorsal height 50% length/total foot length; **BAIT**: Bony arch index truncated = navicular tubercle height/truncated foot length; **ICC**: intraclass correlation coefficient.

**Table A4:** Differences in medial longitudinal arch morphology according to gender.

Measurements		Left Foot			Right Foot		
		Men	Women	*p*	Men	Women	*p*
		Mean ± SD	Mean ± SD		Mean ± SD	Mean ± SD	
**FPI-6**		2.48 ± 4.87	3.85 ± 4.54	0.004	2.62 ± 5.02	3.72 ± 4.23	0.021
**Navicular tubercle height**		4.35 ± 0.80	3.75 ± 0.67	<0.001	4.52 ± 0.8	3.89 ± 0.62	<0.001
**Total foot length**		26.38 ± 1.28	24.06 ± 1.09	<0.001	26.36 ± 1.30	24.06 ± 1.08	<0.001
**Truncated foot length**		19.13 ± 1.00	17.46 ± 0.90	<0.001	19.23 ± 1.04	17.58 ± 0.89	<0.001
**Dorsal height 50% length**		6.24 ± 0.51	5.50 ± 0.519	<0.001	6.25 ± 0.52	5.54 ± 0.45	<0.001
**BAI**		0.16 ± 0.03	0.15 ± 0.03	0.001	0.17 ± 0.03	0.16 ± 0.03	0.001
**AHI**		0.24 ± 0.02	0.23 ± 0.02	<0.001	0.24 ± 0.02	0.23 ± 0.02	0.001
**BAIT**		0.23 ± 0.04	0.21 ± 0.04	0.002	0.24 ± 0.04	0.22 ± 0.04	<0.001
**Arch index**		0.24 ± 0.05	0.24 ± 0.06	0.226	0.25 ± 0.05	0.24 ± 0.06	0.062
**SCORES**		***n* (%)**	***n* (%)**	***p***	***n* (%)**	***n* (%)**	***p***
**FPI-6**	**Normal (0–+5)**	60 (38.5)	115 (43.2)	0.072	65 (41.4)	123 (46.2)	0.015
**Pronated (+6–+9)**		43 (27.6)	75 (28.2)		46 (29.3)	76 (28.6)	
**Highly pronated (+10–+12)**		12 (7.7)	31 (11.7)		9 (5.7)	24 (9.0)	
**Supinated (−1–−4)**		26 (16.7)	35 (13.2)		21 (13.4)	36 (13.5)	
**Highly supinated (−5–−12)**		15 (9.6)	10 (3.8)		16 (10.2)	7 (2.6)	
**AI**	**Normal arch (0.21–0.26)**	73 (46.5)	90 (34.0)	0.017	66 (42.0)	118 (44.5)	0.046
**Flat arch (>0.26)**		52 (33.1)	93 (35.1)		65 (41.4)	82 (30.9)	
**Cavus arch (<0.21)**		32 (20.4)	82 (30.9)		26 (16.6)	65 (24.5)	

**BAI**: bony arch index = navicular tubercle height/total foot length; **AHI**: arch height index = dorsal height 50% length/total foot length; **BAIT**: bony arch index truncated = navicular tubercle height/truncated foot length.

**Table A5:** Differences in medial longitudinal arch morphology according to body mass index.

Measurements	Left Foot					Right Foot				
	Normal Weight	Over Weight	Obesity			Normal Weight	Over Weight	Obesity		
**MEN**	**Mean ± SD**	**Mean ± SD**	**Mean ± SD**	***p***	**BMI r (*p*)**	**Mean ± SD**	**Mean ± SD**	**Mean ± SD**	***p***	**BMI r (*p*)**
**FPI-6**	1.74 ± 4.74	2.86 ± 5.01	2.68 ± 4.79	0.424	0.085 (0.293)	1.76 ± 5.31	3.06 ± 4.73	2.83 ± 5.17	0.386	0.093 (0.249)
**BAI**	0.17 ± 0.03	0.17 ± 0.03	O.16 ± 0.03	0.986	0.003 (0.973)	0.17 ± 0.02	0.16 ± 0.03	0.16 ± 0.03	0.836	−0.017 (0.834)
**AHI**	0.23 ± 0.02	0.24 ± 0.02	0.24 ± 0.02	0.395	0.099 (0.217)	0.24 ± 0.02	0.24 ± 0.02	0.24 ± 0.02	0.412	0.099 (0.218)
**BAIT**	0.23 ± 0.04	0.23 ± 0.05	0.23 ± 0.05	0.981	0.019 (0.816)	0.23 ± 0.03	0.22 ± 0.04	0.21 ± 0.04	0.863	−0.017 (0.834)
**Arch index**	0.23 ± 0.05	0.24 ± 0.05	0.26 ± 0.05	0.030	0.201 (0.011)	0.22 ± 0.05	0.25 ± 0.06	0.28 ± 0.05	0.019	0.187 (0.019)
**AI SCORE**	***n* (%)**	***n* (%)**	***n* (%)**	***p***		***n* (%)**	***n* (%)**	***n* (%)**	***p***	
**Normal arch (0.21–0.26)**	20 (43.5)	34 (48.6)	19 (46.3)	0.487		23 (50.0)	29 (41.4)	14 (34.1)	0.067	
**Flat arch (>0.26)**	14 (30.4)	21 (30.0)	17 (41.5)			12 (26.1)	30 (42.9)	23 (56.1)		
**Cavus arch (<0.21)**	12 (26.1)	15 (21.4)	5 (12.2)			11 (23.9)	11 (15.7)	4 (9.8)		
**WOMEN**	**Mean ± SD**	**Mean ± SD**	**Mean ± SD**	***p***	**BMI r (*p*)**	**Mean ± SD**	**Mean ± SD**	**Mean ± SD**	***p***	**BMI r (*p*)**
**FPI-6**	3.89 ± 4.26	3.70 ± 4.74	4.03 ± 4.91	0.772	0.007 (0.913)	3.81 ± 4,08	3.62 ± 4,45	3.69 ± 4,33	0.961	−0.015 (0.806)
**BAI**	0.16 ± 0.03	0.15 ± 0.03	0.15 ± 0.03	0.023	−0.160 (0.009)	0.17 ± 0.02	0.16 ± 0.03	0.15 ± 0.03	0.016	−0.162 (0.008)
**AHI**	0.23 ± 0.02	0.24 ± 0.02	0.24 ± 0.02	0.496	−0.060 (0.329)	0.23 ± 0.02	0.23 ± 0.02	0.23 ± 0.02	0.879	0.016 (0.790)
**BAIT**	0.22 ± 0.04	0.21 ± 0.04	0.21 ± 0.05	0.021	−0.161 (0.009)	0.23 ± 0.03	0.22 ± 0.04	0.21 ± 0.04	0.012	−0.168 (0.006)
**Arch Index**	0.21 ± 0.06	0.25 ± 0.06	0.28 ± 0.04	<0.001	0.463 (<0.001)	0.22 ± 0.05	0.25 ± 0.06	0.28 ± 0.05	<0.001	0.441 (<0.001)
**AI SCORE**	***n* (%)**	***n* (%)**	***n* (%)**	***p***		***n* (%)**	***n* (%)**	***n* (%)**	***p***	
**Normal arch (0.21–0.26)**	43 (35.0)	29 (37.7)	17 (26.6)	<0.001		64 (52.0)	32 (41.6)	21 (32.8)	<0.001	
**Flat arch (>0.26)**	22 (17.9)	31 (40.3)	40 (62.5)			17 (13.8)	28 (36.4)	37 (57.8)		
**Cavus arch (<0.21)**	58 (47.2)	17 (22.1)	7 (10.9)			42 (34.1)	17 (22.1)	6 (9.4)		

**BAI**: bony arch index = navicular tubercle height/total foot length; **AHI**: arch height index = dorsal height 50% length/total foot length; **BAIT**: bony arch index truncated = navicular tubercle height/truncated foot length. Underweight: BMI ≤ 18.5 kg/m^2^; normal weight: 18.5 kg/m^2^ ≤ BMI < 25 kg/m^2^; overweight: 25 kg/m^2^ ≤ BMI < 30 kg/m^2^; obesity ≥ 30 kg/m^2^.

**Table A6:** Differences and correlation in medial longitudinal arch morphology according to body mass index by gender.

Measurements	Left Foot					Right Foot				
	Normal Weight	Over Weight	Obesity			Normal Weight	Over Weight	Obesity		
**MEN**	**Mean ± SD**	**Mean ± SD**	**Mean ± SD**	***p***	**BMI r (*p*)**	**Mean ± SD**	**Mean ± SD**	**Mean ± SD**	***p***	**BMI r (*p*)**
**FPI-6**	1.74 ± 4.74	2.86 ± 5.01	2.68 ± 4.79	0.424	0.085 (0.293)	1.76 ± 5.31	3.06 ± 4.73	2.83 ± 5.17	0.386	0.093 (0.249)
**BAI**	0.17 ± 0.03	0.17 ± 0.03	0.16 ± 0.03	0.986	0.003 (0.973)	0.17 ± 0.02	0.16 ± 0.03	0.16 ± 0.03	0.836	−0.017 (0.834)
**AHI**	0.23 ± 0.02	0.24 ± 0.02	0.24 ± 0.02	0.395	0.099 (0.217)	0.24 ± 0.02	0.24 ± 0.02	0.24 ± 0.02	0.412	0.099 (0.218)
**BAIT**	0.23 ± 0.04	0.23 ± 0.05	0.23 ± 0.05	0.981	0.019 (0.816)	0.23 ± 0.03	0.22 ± 0.04	0.21 ± 0.04	0.863	−0.017 (0.834)
**Arch index**	0.23 ± 0.05	0.24 ± 0.05	0.26 ± 0.05	0.030	0.201 (0.011)	0.22 ± 0.05	0.25 ± 0.06	0.28 ± 0.05	0.019	0.187 (0.019)
**AI SCORE**	***n* (%)**	***n* (%)**	***n* (%)**	***p***		***n* (%)**	***n* (%)**	***n* (%)**	***p***	
**Normal arch (0.21–0.26)**	20 (43.5)	34 (48.6)	19 (46.3)	0.487		23 (50.0)	29 (41.4)	14 (34.1)	0.067	
**Flat arch (>0.26)**	14 (30.4)	21 (30.0)	17 (41.5)			12 (26.1)	30 (42.9)	23 (56.1)		
**Cavus arch (<0.21)**	12 (26.1)	15 (21.4)	5 (12.2)			11 (23.9)	11 (15.7)	4 (9.8)		
**WOMEN**	**Mean ± SD**	**Mean ± SD**	**Mean ± SD**	***p***	**BMI r (*p*)**	**Mean ± SD**	**Mean ± SD**	**Mean ± SD**	***p***	**BMI r (*p*)**
**FPI-6**	3.89 ± 4.26	3.70 ± 4.74	4.03 ± 4.91	0.772	0.007 (0.913)	3.81 ± 4.08	3.62 ± 4.45	3.69 ± 4.33	0.961	−0.015 (0.806)
**BAI**	0.16 ± 0.03	0.15 ± 0.03	0.15 ± 0.03	0.023	−0.160 (0.009)	0.17 ± 0.02	0.16 ± 0.03	0.15 ± 0.03	0.016	−0.162 (0.008)
**AHI**	0.23 ± 0.02	0.24 ± 0.02	0.24 ± 0.02	0.496	−0.060 (0.329)	0.23 ± 0.02	0.23 ± 0.02	0.23 ± 0.02	0.879	0.016 (0.790)
**BAIT**	0.22 ± 0.04	0.21 ± 0.04	0.21 ± 0.05	0.021	−0.161 (0.009)	0.23 ± 0.03	0.22 ± 0.04	0.21 ± 0.04	0.012	−0.168 (0.006)
**Arch Index**	0.21 ± 0.06	0.25 ± 0.06	0.28 ± 0.04	<0.001	0.463 (<0.001)	0.22 ± 0.05	0.25 ± 0.06	0.28 ± 0.05	<0.001	0.441 (<0.001)
**AI SCORE**	***n* (%)**	***n* (%)**	***n* (%)**	***p***		***n* (%)**	***n* (%)**	***n* (%)**	***p***	
**Normal arch (0.21–0.26)**	43 (35.0)	29 (37.7)	17 (26.6)	<0.001		64 (52.0)	32 (41.6)	21 (32.8)	<0.001	
**Flat arch (>0.26)**	22 (17.9)	31 (40.3)	40 (62.5)			17 (13.8)	28 (36.4)	37 (57.8)		
**Cavus arch (<0.21)**	58 (47.2)	17 (22.1)	7 (10.9)			42 (34.1)	17 (22.1)	6 (9.4)		

**BAI**: bony arch index = navicular tubercle height/total foot length; **AHI**: arch height index = dorsal height 50% length/total foot length; **BAIT**: bony arch index truncated = navicular tubercle height/truncated foot length. Underweight: BMI ≤ 18.5 kg/m^2^; normal weight: 18.5 kg/m^2^ ≤ BMI < 25 kg/m^2^; overweight: 25 kg/m^2^ ≤ BMI < 30 kg/m^2^; obesity ≥ 30 kg/m^2^.
